# Megacity-centric mass mobility during Eid holidays: a unique concern for infectious disease transmission in Bangladesh

**DOI:** 10.1186/s41182-022-00417-4

**Published:** 2022-03-24

**Authors:** Mohammad Sorowar Hossain

**Affiliations:** 1 Department of Emerging and Infectious Diseases, Biomedical Research Foundation, Dhaka, Bangladesh; 2 School of Environment and Life Science, Independent University, Dhaka, Bangladesh

## Abstract

Human mobility, particularly during certain festivals in rapidly growing megacities in low- and middle-income countries, has critical implications in infectious diseases surveillance and preparedness. In this perspective, we present the interesting case of Dhaka megacity, the capital of Bangladesh with a population of over 20 million. In recent times, three massive infectious disease outbreaks in Dhaka (chikungunya, dengue and COVID-19) coincided with Muslim religious Eid festivals. From a public health standpoint, it is very important to share this information with the international community to fight against emerging infectious diseases around the world.

Dear editor

Densely populated cities are uniquely vulnerable to infectious disease outbreaks and its spread because of intense local and global connectivity. Understanding the dynamics of human mobility in the context of rapidly growing megacities in low- and middle-income countries, particularly during certain festivals, has critical implications in surveillance and preparedness. Here we present the case of Dhaka megacity, the capital of Bangladesh (~ 165 million population), in connection to recent major disease outbreaks.

With nearly 20 million inhabitants, Dhaka is the most crowded (spatial density of 47,400/sq km) megacity in the world [1]. This is the main destination of the country’s two-thirds of underprivileged internal migrants from rural areas. Annually, 400,000 low-income climate migrants are also added to the city [2]. Unlike megacities in developed countries, Dhaka is a unique city serving as the main center for the county’s all business, administrative, healthcare facilities, and educational activities. Interestingly, a unique mass movement event happens twice every year when about 10–12 million inhabitants leave Dhaka city to celebrate Eid, the biggest Muslim festival, across the country [3, 4].

In recent times, three massive infectious disease outbreaks in Dhaka (chikungunya, dengue and COVID-19) coincided with the Eid festivals. In 2017, the first-ever chikungunya outbreak devastated Dhaka with an estimated incidence of 47/1000 (translates into a few hundred thousand) [5, 6]. Over 4 million mobile subscribers left the city during the peak of the outbreak to celebrate Eid festival. As expected, the outbreak had spread to other parts of the country [5]. This megacity again confronted the most severe dengue outbreak in 2019 since the first reported outbreak in 2000 and over 100,000 patients were hospitalized. Before Eid, the major proportion of cases was reported from Dhaka as compared to the rest of the country. However, this trend was reversed after the Eid exodus. We found that the incidence of dengue cases decreased sharply (around 24-fold) in Dhaka after the Eid festival, while a net increase (4- to 7-fold) was observed in some peripheral districts [4].

Unlike respiratory infectious diseases (such COVID-19), a nexus of pathogen, vector, and human is required for the transmission of dengue virus from endemic to non-endemic regions. Therefore, the transmission of dengue and chikungunya is much slower. It is important to mention here that homogeneous climatic conditions and presence of *Aedes* mosquitos across Bangladesh may promote dengue transmission rapidly when outbreaks coincide with mass movement events. In 2014–2015, a survey on dengue vectors found that *Aedes* mosquitoes are adapted to non-endemic regions of Bangladesh: the higher prevalence of *Ae. aegypti* documented in urban areas while *Ae. albopictus* was more abundant in rural areas across Bangladesh [7]. Climatic factors including temperature and rainfall typically do not vary significantly across the country [8]. Therefore, an increase in the migration of dengue patients and the carriers from Dhaka to other non-endemic regions during the 2019 outbreak could have facilitated the transmission. This is in line with a recently published paper where it is reported that during 2019 massive dengue outbreak, over 40% of hospitalized dengue cases in a northwestern region had not had a travel history to Dhaka, the epicenter of the outbreak [9].

Bangladesh also struggled with the worst phase of COVID-19 pandemic. The military was deployed to impose a strict lockdown. Nonetheless, over 10 million mobile subscribers left the city to celebrate the Eid festival while the restriction was eased for a week (15–22 July 2021) [10]. Similar Eid exodus happened in the first phase of COVID-19 pandemic in 2020 [11] and May 2021 while testing positivity rate was nearly 10%; current test positivity rate is around 30%. Indonesia’s recent COVID-19 onslaught has also been attributed to Eid exodus [12]. Millions of Muslims around the world engage in internal mass mobility and gatherings during Eid holidays. Mass migration during the Chinese New Year is also linked with the spread of COVID-19 pandemic from Wuhan [13].

Effective public health communication is critical for the prevention of infectious disease outbreaks. Most importantly, building trust among communities is the cornerstone of public health risk communication and intervention. Given the fact that religion plays an important role in the society of Bangladesh and religious leaders are highly respected in the community. Therefore, engaging religious leaders, particularly imam of mosques along with trustworthy public figures and eminent researchers would be instrumental for managing infectious disease outbreaks, particularly during major religious holidays.

In a highly interconnected world, mass movement during religious festivals is a matter of great concern, if it coincides with an outbreak—especially in low- and middle-income countries where resources are inadequate for managing infectious disease outbreaks. In Bangladesh, the infectious disease surveillance system is not well established due to the lack of an organized medical record-keeping system and other resources. For instance, even though Dhaka is hyper-endemic for dengue, an information-based decision-making system (robust surveillance of hospitalized cases and monitoring of molecular evolution of genotypes of dengue) has not been established yet [14]. We, therefore, call for international cooperation and collaboration to establish a task force for the surveillance of emerging infectious diseases worldwide.

## Data Availability

None.

## Abbreviation

COVID-19: Coronavirus diseases
